# Association of *OPG* gene polymorphisms with the risk of knee osteoarthritis among Chinese people

**DOI:** 10.1002/mgg3.662

**Published:** 2019-04-11

**Authors:** Yuxin Qi, Feimeng An, Jiaqi Wang, Yuan Liu, Hongyan Gao, Zhe Ge, Enze Jiang, Donggao Cai, Jianping Shi, Jianzhong Wang

**Affiliations:** ^1^ Inner Mongolia Medical University Hohhot Inner Mongolia China; ^2^ The 2nd affiliated Hospital of Inner Mongolia Medical University Hohhot Inner Mongolia China; ^3^ Department of Orthopaedics Jinshan Hospital, Fudan University Shanghai City China; ^4^ Shanghai Changzheng hospital Shanghai China; ^5^ Shengjing Hospital affiliated to China Medical University Shenyang China

**Keywords:** Chinese people, gene polymorphisms, knee osteoarthritis, *OPG*

## Abstract

**Background:**

Osteoarthritis (OA) is usually recognized to have a genetic factor, and in our study, we performed a case–control study to analyze the association between 14 single nucleotide polymorphisms (SNPs) in *OPG* and the risk of knee OA in a Chinese Han population.

**Methods:**

Fourteen OPG SNPs were assayed using MassARRAY in 393 patients clinically and radiographically diagnosed with knee OA and in 500 controls. Allelic and genotypic frequencies were compared between the groups. Logistic regression adjusting for age and gender was used to estimate risk associations between specific genotypes and knee OA by computing odds ratios (ORs) and 95% confidence intervals (95% CIs).

**Results:**

We found that the minor alleles of six SNPs in OPG were associated with an increased or decreased risk of knee OA in the allelic model analysis. In the genetic model analysis, we found that rs1905786, rs1032128, rs3134058, rs11573828, rs11573849, rs3134056, and rs1564861 were associated with an increased or decreased risk of knee OA before adjusted by sex and age. And after adjustment, three SNPs (rs1485286, rs1905786, and rs1032128) were identified to have a negative effect on knee OA.

**Conclusion:**

Our results verify that genetic variants of *OPG* contribute to knee OA susceptibility in the population of northern China. These genetic associations may identify individuals at a particularly high risk of developing knee OA.

## Abstract 2

Osteoarthritis (OA) is usually recognized to have a genetic factor, and in our study, we performed a case–control study to analyze the association between 14 single nucleotide polymorphisms (SNPs) in OPG and the risk of knee OA in a Chinese Han population. Our results verify that genetic variants of OPG contribute to knee OA susceptibility in the population of northern China. These genetic associations may identify individuals at a particularly high risk of developing knee OA.

## 1 INTRODUCTION

Osteoarthritis (OA) is a kind of chronic joint disease which is characterized by the progressive destruction of the cartilage matrix and bony changes (Karsdal et al., 2008). The occurrence of knee OA is a complex process involving a variety of risk factors, including genetic predisposition, age, obesity, inflammation, and pre‐overload activity. The incidence of knee OA is pretty high and it is not easy to have an early effective treatment. It has become a serious burden to the family and the whole society. However, the pathogenesis of this disease has not been fully defined yet. It is urgent to define the pathogenesis of knee OA so as to prevent and reduce the occurrence of knee OA effectively.

It is well known that some members of the tumor necrosis factor (TNF) family play a significant role in regulating bone metabolism. In this context, a molecular triad made up of OPG/RANK/RANKL has been lately described as being the essential cytokine system for controlling the differentiation and function of osteoclast biology (Kearns, Khosla, & Kostenuik, 2008). RANKL (receptor activator of nuclear factorκB ligand) (Suda et al., 1999) is a member of the TNF ligand superfamily that regulates bone metabolism. It interacts with its receptor, RANK (receptor activator of nuclear factorκB) expressed on osteoclast precursors to induce differentiation and activation of osteoclasts (Hofbauer & Heufelder, 2000; Jones, Kong, & Penninger, 2002; Khosla, 2002). Osteoprotegerin (OPG) is a soluble decoy receptor for RANKL preventing it from binding to its receptor RANK (Boyce & Xing, 2007; Jones et al., 2002; Khosla, 2002). OPG, by interacting with RANKL, inhibits the binding of RANKL to RANK, thereby preventing RANK activation and subsequent osteoclastogenesis and, as a result, inhibiting bone resorption (Gravallese & Goldring, 2000). The structural integrity of articular cartilage is thought to be influenced by changes in subchondral bone (Felson & Neogi, 2004; Hayami et al., 2004) with denser bone being seen below OA cartilage. Both RANKL and OPG are expressed and synthesized by articular chondrocytes (Komuro et al., 2001; Kwan Tat et al., 2009; Moreno‐Rubio, Herrero‐Beaumont, Tardío, & Largo, 2007) and in a position adjacent to subchondral bone, these cytokines might influence bone turnover and cause changes in bone density.

So far, few relevant reports have shown that the association of *OPG* gene polymorphisms with the risk of osteoarthritis. In this study, we performed a case–control study to analyze the association between 14 single nucleotide polymorphisms (SNPs) in *OPG* and the risk of knee OA in a Chinese Han population. This study will have an important role in the understanding of the disease pathogenesis and the prediction of the prognosis of knee OA. A good basis will be provided for other scholars of osteoarthritis research.

## 2 PATIENTS AND METHODS

### 2.1 Ethics statement

The protocol in this study was completed in accordance with the principles of the Declaration of Helsinki and was ratifed by the ethics committee of the International Mongolia Medicine Hospital. The participants all provided signed informed consent.

### 2.2 Subjects

A total of 393 Han Chinese patients with knee OA (132 men and 261 women) were recruited from February 2014 to July 2016 at the Second Affiliated Hospital, Inner Mongolia Medical University. Simultaneously, 500 healthy, unrelated controls without signs or symptoms of OA were recruited based on a medical examination at the same hospital. All of the chosen subjects were from the Second Affiliated Hospital, Inner Mongolia Medical University. To reduce the potential environmental and therapeutic factors impacting the variation of complex human diseases, we performed detailed recruitment and set exclusion criteria to exclude subjects with diseases related to genetic susceptibility, such as tumor.

The diagnosis of knee OA was based on a detailed history, physical exam, and/or radiographic studies. Diagnostic criteria developed by the American College of Rheumatology (ACR) include the presence of knee joint pain, osteophytes or bone spurs on X‐ray, and one or more associated symptoms in the knee joint. This research was approved by the ethics committee of Inner Mongolia Medical University and written informed consent was gained from all participants.

### 2.3 SNP selection and genotyping

For *OPG* gene, all tagging SNPs were selected from the HapMap database (http://www.hapmap.org/). Fourteen SNPs which had minor allele frequencies >5% in Asians were finally selected for genotyping. All of these SNPs can be authenticated by the NCBI (http://www.ncbi.nlm.nih.gov/SNP/) and HapMap databases. Five‐milliliters of blood samples were collected in ethylenediamine‐tetra‐acetic acid (EDTA) containing tubes and were stored at −20°C. DNA was isolated by the GoldMag extraction method (GoldMag Co. Ltd, Xi'an, China). The Sequenom MassARRAY Assay Design 3.0 Software (San Diego, CA) was used to design Multiplexed SNP MassEXTEND assays (Gabriel, Ziaugra, & Tabbaa, 2009). A Sequenom MassARRAY RS1000 was used to perform the SNP genotyping according to the manufacturer's protocol.

### 2.4 Statistical methods

All of the statistical analyses were performed with the SPSS 19.0 software for Windows (SPSS, Chicago, IL). Allele and genotype frequencies were obtained by direct counts. Hardy–Weinberg equilibrium for each SNP was determined using an exact test to compare the expected frequencies of genotypes in controls. A probability value *p* less than 0.05 was considered statistically significant. Allele and genotype frequencies in knee OA groups and controls were calculated by chi‐squared test/Fisher's exact tests. Five genetic models (codominant, dominant, recessive, over‐dominant, and additive) were applied by PLINK software (http://pngu.mgh.harvard.edu/purcell/plink/). Odds ratios (ORs) and 95% confidence intervals (CIs) were calculated by unconditional logistic regression analysis, adjusted for age and gender. Ultimately, the Haploview software package (version 4.2) and SHEsis software platform (http:// www.nhgg.org/analysis/) were used to estimate pairwise linkage disequilibrium (LD), haplotype construction, and genetic association at polymorphism loci.

## 3 RESULTS

### 3.1 Participant characteristics

The demographic characteristics of the study population containing gender and age are summarized in Table 1. A total of 393 cases (261 females and 132 males) and 500 controls (103 females and 397 males) were recruited for our study. The mean age of the patients and the control group were 60.89 ± 4.778 years and 47.37 ± 9.915 years, respectively. There were significant differences between patients and controls in age (*p* < 0.05) and gender (*p* < 0.05), and we would adjust the factor in the following analysis.

**Table 1:** Characteristics of cases and controls in this study

Variable	Cases(*n* = 393)	Controls(*n* = 500)	*p* value
Sex			<0.001
Male	132	397	
Female	261	103	
Age, year(mean ± *SD*)	60.89 ± 4.78	47.37 ± 9.92	<0.001

Note

*p* values were calculated from Pearson's chi‐squared test.

### 3.2 Hardy–Weinberg equilibrium and SNP alleles

The basic information about all the SNPs including chromosome, position, alleles, minor allele frequency, and HWE results are shown in Table 2. All of the 14 tag SNPs were in HWE among the control subjects (*p* > 0.05).

**Table 2:** Examined SNPs examined in the OPG gene

SNP	Chromosome	Position	Allele	Minor allele frequency		HWE *p* value	OR (95% CI)	*p* a
				Case	Control			
rs10955911	8	119937741	A/G	0.116	0.134	0.4395	0.846 (0.637–1.124）	0.249
rs3134053	8	119946141	T/C	0.313	0.294	0.3725	1.096 (0.891–1.347）	0.387
rs11573896	8	119947430	A/T	0.125	0.133	0.6969	0.930 (0.703–1.229)	0.606
rs1485286	8	119950668	T/C	0.436	0.381	0.3429	1.258 (1.040–1.522)	0.018*
rs3102725	8	119951005	A/G	0.151	0.155	1	0.973 (0.750–1.261)	0.834
rs1905786	8	119951692	T/C	0.311	0.242	0.9029	1.415 (1.148–1.745)	0.001*
rs1032128	8	119951773	G/A	0.439	0.382	0.1557	1.267 (1.048–1.532)	0.015*
rs3134056	8	119952212	G/A	0.368	0.440	0.7857	0.740 (0.611–0.896)	0.002*
rs3134058	8	119954108	G/A	0.472	0.410	0.7816	1.287 (1.066–1.554)	0.009*
rs11573856	8	119954995	A/G	0.149	0.175	0.2161	0.822 (0.637–1.062)	0.133
rs11573849	8	119956378	T/G	0.160	0.151	0.1616	1.073 (0.830–1.389)	0.59
rs3102731	8	119959389	A/G	0.152	0.157	1	0.961 (0.742–1.245)	0.762
rs11573828	8	119959813	T/C	0.161	0.147	0.213	1.107 (0.854–1.435)	0.442
rs1564861	8	119965909	C/A	0.369	0.449	0.7173	0.718 (0.593–0.869)	0.00066*

Note

95% CI: 95% confidence interval; HWE: Hardy–Weinberg equilibrium; OR: odds ratio.

a *p* values were calculated from chi‐squared test/Fisher's exact test.

* *p* ≤ 0.05 indicates statistical significance.

### 3.3 Association between *OPG*polymorphisms and knee OA risk

Genetic models (codominant, dominant, recessive, over‐dominant, and log‐additive) and the genotype frequencies were used to further identify the associations between the OPG SNPs and the risk of knee OA (Table 3).

**Table 3:** Association between SNPs and knee OA in multiple inheritance models

SNP	Model	Genotype	Group = control	Group = case	Without adjustment		With adjustment	
					OR (95% CI)	*p* value	OR (95% CI)	*p* value
rs1485286	Codominant	C/C	186 (37.3%)	131 (33.4%)	1	0.016*	1	0.026*
		C/T	246 (49.3%)	180 (45.9%)	1.04 (0.77–1.40)		0.97 (0.61–1.54)	
		T/T	67 (13.4%)	81 (20.7%)	1.72 (1.16–2.54)		2.13 (1.14–3.98)	
	Dominant	C/C	186 (37.3%)	131 (33.4%)	1	0.23	1	0.41
		C/T‐T/T	313 (62.7%)	261 (66.6%)	1.18 (0.90–1.56)		1.20 (0.78–1.85)	
	Recessive	C/C‐C/T	432 (86.6%)	311 (79.3%)	1	0.0041*	1	0.007*
		T/T	67 (13.4%)	81 (20.7%)	1.68 (1.18–2.39)		2.16 (1.22–3.83)	
	Overdominant	C/C‐T/T	253 (50.7%)	212 (54.1%)	1	0.32	1	0.22
		C/T	246 (49.3%)	180 (45.9%)	0.87 (0.67–1.14)		0.77 (0.50–1.17)	
	Log‐additive	–	–	–	1.26 (1.04–1.52)	0.018*	1.35 (1.00–1.82)	0.046*
rs1905786	Codominant	C/C	288 (57.6%)	195 (49.7%)	1	0.0028*	1	0.077
		C/T	182 (36.4%)	150 (38.3%)	1.22 (0.92–1.61)		1.19 (0.76–1.85)	
		T/T	30 (6%)	47 (12%)	2.31 (1.41–3.79)		2.49 (1.11–5.60)	
	Dominant	C/C	288 (57.6%)	195 (49.7%)	1	0.019*	1	0.16
		C/T‐T/T	212 (42.4%)	197 (50.3%)	1.37 (1.05–1.79)		1.35 (0.89–2.06)	
	Recessive	C/C‐C/T	470 (94%)	345 (88%)	1	0.0016*	1	0.033*
		T/T	30 (6%)	47 (12%)	2.13 (1.32–3.44)		2.32 (1.05–5.12)	
	Overdominant	C/C‐T/T	318 (63.6%)	242 (61.7%)	1	0.57	1	0.8
		C/T	182 (36.4%)	150 (38.3%)	1.08 (0.82–1.42)		1.06 (0.69–1.63)	
	Log‐additive	–	–	–	1.39 (1.13–1.71)	0.0016*	1.40 (1.01–1.94)	0.045*
rs1032128	Codominant	A/A	183 (36.7%)	130 (33.1%)	1	0.0076*	1	0.023*
		A/G	251 (50.3%)	181 (46.1%)	1.02 (0.76–1.36)		0.99 (0.62–1.56)	
		G/G	65 (13%)	82 (20.9%)	1.78 (1.20–2.64)		2.18 (1.17–4.09)	
	Dominant	A/A	183 (36.7%)	130 (33.1%)	1	0.26	1	0.37
		A/G‐G/G	316 (63.3%)	263 (66.9%)	1.17 (0.89–1.55)		1.22 (0.79–1.88)	
	Recessive	A/A‐A/G	434 (87%)	311 (79.1%)	1	0.0018*	1	0.006*
		G/G	65 (13%)	82 (20.9%)	1.76 (1.23–2.51)		2.20 (1.24–3.90)	
	Overdominant	A/A‐G/G	248 (49.7%)	212 (53.9%)	1	0.21	1	0.22
		A/G	251 (50.3%)	181 (46.1%)	0.84 (0.65–1.10)		0.77 (0.51–1.17)	
	Log‐additive	–	–	–	1.27 (1.05–1.54)	0.014*	1.37 (1.01–1.84)	0.039*
rs3134056	Codominant	A/A	155 (31%)	158 (40.2%)	1	0.0078*	1	0.89
		A/G	250 (50%)	181 (46.1%)	0.71 (0.53–0.95)		0.89 (0.56–1.41)	
		G/G	95 (19%)	54 (13.7%)	0.56 (0.37–0.83)		0.94 (0.50–1.77)	
	Dominant	A/A	155 (31%)	158 (40.2%)	1	0.0043*	1	0.65
		A/G‐G/G	345 (69%)	235 (59.8%)	0.67 (0.51–0.88)		0.90 (0.59–1.40)	
	Recessive	A/A‐A/G	405 (81%)	339 (86.3%)	1	0.035*	1	0.99
		G/G	95 (19%)	54 (13.7%)	0.68 (0.47–0.98)		1.00 (0.57–1.78)	
	Overdominant	A/A‐G/G	250 (50%)	212 (53.9%)	1	0.24	1	0.65
		A/G	250 (50%)	181 (46.1%)	0.85 (0.66–1.11)		0.91 (0.60–1.38)	
	Log‐additive	–	–	–	0.74 (0.61–0.90)	0.002*	0.95 (0.71–1.29)	0.76
rs3134058	Codominant	A/A	172 (34.5%)	121 (30.8%)	1	0.0056*	1	0.73
		A/G	245 (49.1%)	173 (44%)	1.00 (0.74–1.36)		0.95 (0.59–1.52)	
		G/G	82 (16.4%)	99 (25.2%)	1.72 (1.18–2.49)		1.18 (0.66–2.12)	
	Dominant	A/A	172 (34.5%)	121 (30.8%)	1	0.24	1	0.94
		A/G‐G/G	327 (65.5%)	272 (69.2%)	1.18 (0.89–1.57)		1.02 (0.65–1.58)	
	Recessive	A/A‐A/G	417 (83.6%)	294 (74.8%)	1	0.0013*	1	0.45
		G/G	82 (16.4%)	99 (25.2%)	1.71 (1.23–2.38)		1.22 (0.73–2.04)	
	Overdominant	A/A‐G/G	254 (50.9%)	220 (56%)	1	0.13	1	0.58
		A/G	245 (49.1%)	173 (44%)	0.82 (0.63–1.06)		0.89 (0.59–1.35)	
	Log‐additive	–	–	–	1.27 (1.06–1.53)	0.01*	1.07 (0.81–1.43)	0.63
rs11573828	Codominant	C/C	356 (71.9%)	282 (71.9%)	1	0.036*	1	0.13
		C/T	132 (26.7%)	94 (24%)	0.90 (0.66–1.22)		0.75 (0.46–1.21)	
		T/T	7 (1.4%)	16 (4.1%)	2.89 (1.17–7.11)		2.68 (0.71–10.10)	
	Dominant	C/C	356 (71.9%)	282 (71.9%)	1	0.99	1	0.48
		C/T‐T/T	139 (28.1%)	110 (28.1%)	1.00 (0.74–1.34)		0.85 (0.54–1.35)	
	Recessive	C/C‐C/T	488 (98.6%)	376 (95.9%)	1	0.013*	1	0.1
		T/T	7 (1.4%)	16 (4.1%)	2.97 (1.21–7.28)		2.89 (0.77–10.82)	
	Overdominant	C/C‐T/T	363 (73.3%)	298 (76%)	1	0.36	1	0.17
		C/T	132 (26.7%)	94 (24%)	0.87 (0.64–1.18)		0.72 (0.45–1.16)	
	Log‐additive	–	–	–	1.10 (0.85–1.43)	0.45	0.99 (0.66–1.46)	0.94
rs1564861	Codominant	A/A	149 (29.9%)	149 (29.9%)	1	0.0027*	1	0.85
		A/C	252 (50.5%)	252 (50.5%)	0.69 (0.51–0.92)		0.87 (0.55–1.39)	
		C/C	98 (19.6%)	98 (19.6%)	0.52 (0.35–0.78)		0.92 (0.49–1.72)	
	Dominant	A/A	149 (29.9%)	149 (29.9%)	1	0.0017*	1	0.58
		A/C‐C/C	350 (70.1%)	350 (70.1%)	0.64 (0.48–0.85)		0.88 (0.57–1.37)	
	Recessive	A/A‐A/C	401 (80.4%)	401 (80.4%)	1	0.019*	1	0.97
		C/C	98 (19.6%)	98 (19.6%)	0.65 (0.45–0.94)		0.99 (0.56–1.75)	
	Overdominant	A/A‐C/C	247 (49.5%)	247 (49.5%)	1	0.21	1	0.62
		A/C	252 (50.5%)	252 (50.5%)	0.85 (0.65–1.10)		0.90 (0.59–1.36)	
	Log‐additive	–	–	–	0.72 (0.59–0.87)	0.0006*	0.94 (0.69–1.27)	0.69
rs3134053	Codominant	C/C	236 (50.8%)	194 (49.7%)	1	0.4	1	0.077
		C/T	185 (39.8%)	148 (38%)	0.97 (0.73–1.30)		0.60 (0.38–0.95)	
		T/T	44 (9.5%)	48 (12.3%)	1.33 (0.85–2.08)		0.97 (0.48–1.96)	
	Dominant	C/C	236 (50.8%)	194 (49.7%)	1	0.77	1	0.066
		C/T‐T/T	229 (49.2%)	196 (50.3%)	1.04 (0.80–1.36)		0.67 (0.44–1.03)	
	Recessive	C/C‐C/T	421 (90.5%)	342 (87.7%)	1	0.18	1	0.55
		T/T	44 (9.5%)	48 (12.3%)	1.34 (0.87–2.07)		1.22 (0.63–2.39)	
	Overdominant	C/C‐T/T	280 (60.2%)	242 (62%)	1	0.58	1	0.024*
		C/T	185 (39.8%)	148 (38%)	0.93 (0.70–1.22)		0.61 (0.39–0.94)	
	Log‐additive	–	–	–	1.09 (0.89–1.33)	0.41	0.84 (0.62–1.15)	0.28
rs11573849	Codominant	G/G	356 (71.2%)	283 (72%)	1	0.028*	1	0.092
		G/T	137 (27.4%)	94 (23.9%)	0.86 (0.64–1.17)		0.71 (0.44–1.14)	
		T/T	7 (1.4%)	16 (4.1%)	2.88 (1.17–7.08)		2.68 (0.71–10.14)	
	Dominant	G/G	356 (71.2%)	283 (72%)	1	0.79	1	0.36
		G/T‐T/T	144 (28.8%)	110 (28%)	0.96 (0.72–1.29)		0.81 (0.51–1.28)	
	Recessive	G/G‐G/T	493 (98.6%)	377 (95.9%)	1	0.012*	1	0.096
		T/T	7 (1.4%)	16 (4.1%)	2.99 (1.22–7.34)		2.94 (0.78–11.03)	
	Overdominant	G/G‐T/T	363 (72.6%)	299 (76.1%)	1	0.24	1	0.11
		G/T	137 (27.4%)	94 (23.9%)	0.83 (0.61–1.13)		0.68 (0.43–1.10)	
	Log‐additive	–	–	–	1.07 (0.83–1.39)	0.59	0.95 (0.64–1.41)	0.8

Note

CI: confidence interval; OR: odds ratio.

* *p* ≤ 0.05 indicates statistical, significance.

Four SNPs were discovered to be significantly associated with an increased risk of knee OA (rs1485286 T/C, OR = 1.258, 95% CI: 1.040–1.522; rs1032128 G/A, OR = 1.267, 95% CI: 1.048–1.532; rs1905786 T/C, OR = 1.415, 95% CI: 1.148–1.745; rs3134058 G/A, OR = 1.287, 95% CI: 1.066–1.554). Tow SNPs were found to be associated with a decreased risk of knee OA (rs3134056 G/A, OR = 0.740, 95% CI: 0.611–0.896; rs1564861 C/A, OR = 0.718, 95% CI: 0.593–0.869).

After adjustment, these results showed that three SNPs of OPG (rs1485286 TT vs. CC: adjusted OR = 2.13, 95% CI: 1.14–3.98, *p* = 0.026; recessive: adjusted OR = 2.16, 95% CI: 1.18–2.39, *p* = 0.007; additive model: adjusted OR = 1.35, 95% CI: 1.00–1.82, *p* = 0.046; rs1905786 recessive: adjusted OR = 2.32, 95% CI: 1.05–5.12, *p* = 0.033; additive model: adjusted OR = 1.40, 95% CI: 1.01–1.94, *p* = 0.045; rs1032128 GG vs. AA: adjusted OR = 2.18, 95% CI: 1.17–4.09, *p* = 0.023; recessive: adjusted OR = 2.20, 95% CI: 1.24–3.90, *p* = 0.006; additive: adjusted OR = 1.37, 95% CI: 1.01–1.84, *p* = 0.039) had a positive effect on knee OA. Moreover, the rs3134053 (over‐dominant: adjusted OR = 0.61, 95% CI: 0.39–0.94, *p* = 0.024) was identified to have a negative effect on knee OA.

### 3.4 Haplotype analysis

**Figure 1:**
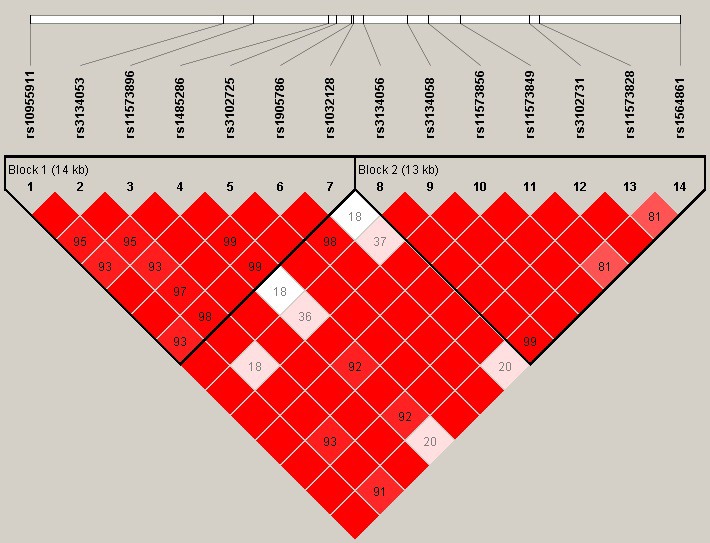
Linkage disequilibrium (LD) of polymorphic sites in the OPG on chromosome 8. The LD pattern was analyzed using the parameters D'

Ultimately, the linkage haplotype construction and disequilibrium were detected and evaluated. Two blocks (Table 4; Figure 1 ) were detected in the OPG SNPs by haplotype analyses. Before adjustment, haplotype “GCTCGCA” was associated with an increased risk of knee OA (OR = 1.71; 95% CI = 1.32–2.23; *p* = 0.0001) in block 1. Meanwhile, haplotype “GCTTGTG” was associated with an increased risk of knee OA (OR = 1.51; 95% CI = 1.10–2.06; *p* = 0.01). In addition, haplotype “ACATGCG” was associated with a significantly increased risk of knee OA (OR = 5.90; 95% CI = 2.11–16.47; *p* = 0.0007). Compared with the “AGGGGCA” wild type, the “GAAGGCC” haplotype was found to be associated with a decreased risk of knee OA in block 2 (OR = 0.67, 95% CI: 0.52–0.86, *p* = 0.0069; OR = 0.66, 95% CI: 0.49–0.89, *p* = 0.092). After adjustment, we also found that haplotype “ACATGCG” was associated with an increased risk of knee OA (OR = 7.51; 95% CI = 1.66–33.88; *p* = 0.0089). Otherwise, haplotype “AGGGACA” was associated with a decreased risk of knee OA (OR = 0.62; 95% CI = 0.39–0.98; *p* = 0.041).

**Table 4:** OPG haplotype frequencies and the association with the risk of knee OA in cases and controls

Block ID	SNPs	Haplotype	Haplotype frequencies		Without adjustment		With adjustment	
			Case	Control	OR (95% CI)	*p* value	OR (95% CI)	*p* value
1	rs10955911, rs3134053, rs11573896, rs1485286, rs3102725, rs1905786, rs1032128	GCTCGCA	0.245	0.317	1.71 (1.32–2.23)	0.0001*	1.44 (0.95–2.19)	0.084
		GCTTGTG	0.307	0.24	1.51 (1.10–2.06)	0.01*	1.15 (0.71–1.87)	0.56
		GTTCGCA	0.159	0.144	1.32 (0.97–1.80)	0.074	0.87 (0.54–1.41)	0.57
		GTTCACA	0.147	0.147	1.13 (0.80–1.57)	0.49	1.03 (0.62–1.72)	0.91
		ACATGCG	0.103	0.103	5.90 (2.11–16.47)	0.0007*	7.51 (1.66–33.88)	0.0089*
		GCATGCG	0.103	0.005	1.26 (0.57–2.80)	0.57	1.68 (0.50–5.70)	0.4
2	rs3134056, rs3134058, rs11573856, rs11573849, rs3102731, rs11573828, rs1564861	AGGGGCA	0.32	0.252	1	0.0021*	1	–
		GAGGGCC	0.219	0.263	0.67 (0.52–0.86)	0.0069*	0.77 (0.52–1.15)	0.21
		GAAGGCC	0.149	0.149	0.66 (0.49–0.89)	0.092*	0.79 (0.50–1.27)	0.33
		AGGGACA	0.152	0.152	0.78 (0.58–1.04)	0.6	0.62 (0.39–0.98)	0.041*
		AAGTGTA	0.159	0.141	0.92 (0.69–1.24)	0.018*	0.84 (0.53–1.32)	0.44

Note

95% CI: 95% confidence interval; OR: odds ratio.

* *p* ≤ 0.05 indicates statistical significance.

## 4 DISCUSSION

In our study, we examined the associations between 14 SNPs of the *OPG* gene and the risk of knee OA. We identified that *OPG* genetic polymorphisms (rs1485286, rs1905786, rs1032128, rs3134056, rs3134058, rs11573828, rs1564861, rs3134053, and rs11573849) are associated with the risk of knee OA in the population of northern China. We also found a strong effect of “GCTCGCA, GCTTGTG and ACATGCG” haplotypes of *OPG* were associated with an increased risk of knee OA. In addition, we also observed that “GAGGGCC and GAAGGCC” haplotypes of knee OA were associated with decreased risk of knee OA.

It has been indicated in our results that there is a statistically significant difference between the knee OA and control groups regarding the *OPG* SNPs, showing a positive association between genetic polymorphism and the susceptibility of knee OA. It is well known that, OPG/RANKL/RANK signaling pathway belongs to the TNF receptor (TNFR) superfamily and also played a crucial role in bone metabolism and osteopathy development, including inherited bone diseases, acquired bone pathologies, osteoarthritis, and osteonecrosis(Di et al., 2015; Kwan Tat et al., 2009; Walsh & Choi, 2014). Furthermore, previous studies performed on mice reported a beneficial effect of OPG against the progression of OA (Kadri et al., 2008; Shimizu et al., 2007).The balance between the expression of RANKL and OPG determines the extent of osteoclast activity and subsequent bone resorption (Kwan Tat et al., 2009; Tat, Pelletier, Velasco, Padrines, & Martel‐Pelletier, 2009). It has also been demonstrated that the ratio of OPG/RANKL in OA chondrocytes is significantly different from those in normal chondrocytes (Pilichou et al., 2008).

In our previous study, we have demonstrated that *OPG* gene polymorphisms (rs1485286, rs1032128, and rs11573828) may increase the risk of alcohol‐induced ONFH and SNP (rs11573856) may be a protective effect (Li et al., 2016). In contrast, we also found that rs1485286 (CM000670.2:g.118938429C>G), rs1032128 (CM000670.2:g.118939534G>A), and rs11573828 (CM000670.2:g.118947574C>T) in *OPG* present same result for the knee OA in the study. In addition, this study has identified rs1905786 (CM000670.2:g.118939453T>A), rs3134058 (CM000670.2:g.118941869G>A), and rs11573849 (CM000670.2:g.118944139G>T) in *OPG* as an increased risk factor in knee OA. Based on this point, OPG was increased in osteoarthritis patients' Synovial (Pilichou et al., 2008), it is understood that alterations in the expression of *OPG* gene may tend to predispose the individual to the generalized development of OA (Shimizu et al., 2007). rs3134056 (CM000670.2:g.118939973A>G), rs1564861(CM000670.2:g.118953670A>C), and rs3134053(CM000670.2:g.118933902T>C) may be a protective effect. In a sense, these SNPs in *OPG* may actually play an important role in biological functions. Subsequent experiments are needed to demonstrate this point. According to previous researches, association of *OPG* gene polymorphisms with the risk of knee OA had been proved in the British population (Valdes et al., 2004,2006). These results were consistent with our findings. Although our targets SNPs are different, but this indicates that OPG may play an important role in the mechanism of osteoarthritis prevention. Accordingly, the biological functions and exact location of the real causal SNPs in *OPG* gene is of great interest and needs further investigation.

In the present study, Haplotype analysis indicated that there was a decrease in significance, but knee OA risk was substantially increased among individuals with specific haplotypes (GCTCGCA haplotype, GCTTGTG haplotype, and ACATGCG haplotype). To sum up, our study provides evidence for associations between the nine SNPs (rs1485286, rs1905786, rs1032128, rs3134056, rs3134058, rs11573828, rs1564861, rs3134053, and rs11573849) of OPG from the OPG/RANK/RANKL system and the risk of knee osteoarthritis. Despite some significant discoveries are revealed in our study, there are still a few limitations (Lin et al., 2017; Liu et al., 2017; Wang et al., 2018). First, our study lacks the part of biological function analysis, which will be crucial for elucidating the role of OPG in knee OA. Second, we also performed Bonferroni correction analysis, but there are a few statistical significant associations between OPG SNPs and knee OA. Nevertheless, this may be due to the weakness of Bonferroni correction itself. Third, we used the case–control design of hospital based, which may cause selection bias. Finally, the sample might convert the positive findings into negative results. To make our conclusions more convincing, a larger case–control study may be expected to solve those problems.

## CONFLICT OF INTEREST

The authors declare that there are no conflict of interest.

5
